# Deciphering the pseudouridine nucleobase modification in human diseases: From molecular mechanisms to clinical perspectives

**DOI:** 10.1002/ctm2.70190

**Published:** 2025-01-20

**Authors:** Shiheng Jia, Xue Yu, Na Deng, Chen Zheng, Mingguang Ju, Fanglin Wang, Yixiao Zhang, Ziming Gao, Yanshu Li, Heng Zhou, Kai Li

**Affiliations:** ^1^ Department of Surgical Oncology and General Surgery The First Hospital of China Medical University Shenyang Liaoning China; ^2^ Department of Hematology The Fourth Affiliated Hospital of China Medical University Shenyang Liaoning China; ^3^ Department of Anesthesiology The First Hospital of China Medical University Shenyang Liaoning China; ^4^ Department of Cell Biology Key Laboratory of Cell Biology National Health Commission of the PRC and Key Laboratory of Medical Cell Biology Ministry of Education of the PRC China Medical University Shenyang Liaoning China; ^5^ Key Laboratory of Molecular Pathology and Epidemiology of Gastric Cancer in Liaoning Education Department The First Hospital of China Medical University Shenyang Liaoning China

**Keywords:** cancer, disease, post‐transcriptional modification, pseudouridylation

## Abstract

RNA pseudouridylation, a dynamic and reversible post‐transcriptional modification found in diverse RNA species, is crucial for various biological processes, including tRNA homeostasis, tRNA transport, translation initiation regulation, pre‐mRNA splicing, enhancement of mRNA translation, and translational fidelity. Disruption of pseudouridylation impairs cellular homeostasis, contributing to pathological alterations. Recent studies have highlighted its regulatory role in human diseases, particularly in tumourigenesis. Cellular stresses trigger RNA pseudouridylation in organisms, suggesting that pseudouridylation‐mediated epigenetic reprogramming is essential for maintaining cellular viability and responding to stress. This review examines the regulatory mechanisms and pathological implications of pseudouridylation in human diseases, with a focus on its involvement in tumourigenesis. Additionally, it explores the therapeutic potential of targeting pseudouridylation, presenting novel strategies for disease treatment.

**Highlights:**

- Methods to detect pseudouridine were introduced from classic mass spectrometry‐based methods to newer approaches such as nanopore‐based technologies and BID sequencing, each with its advantages and limitations.
- RNA pseudouridylation is crucial for various biological processes, including tRNA homeostasis, tRNA transport, translation initiation regulation, pre‐mRNA splicing, enhancement of mRNA translation, and translational fidelity.
- Increased pseudouridylation is frequently associated with tumour initiation, progression, and poor prognosis, whereas its reduction is predominantly implicated in non‐tumour diseases.
- A comprehensive understanding of the inducing factors for RNA pseudouridylation will be essential for elucidating its role in diseases. Such insights can provide robust evidence for how pseudouridylation influences disease progression and offer new avenues for therapeutic strategies targeting pseudouridylation dysregulation.
- The therapeutic potential of RNA pseudouridylation in diseases is enormous, including inhibitors targeting pseudouridine synthases, the application of RNA pseudouridylation in RNA therapeutics, and its role as a biological marker.

## Graphical abstract

This review discusses the regulatory mechanisms and pathological significance of pseudouridylation in human diseases, with a special emphasis on its involvement in tumourigenesis. Furthermore, the potential therapeutic advantages of targeting pseudouridylation are explored, offering novel strategies for disease treatment.

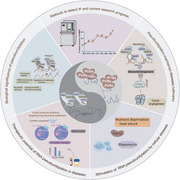

## 1 INTRODUCTION

Over 170 distinct modified nucleosides have been identified to date.^1^ Among them, pseudouridine (Ψ) is one of the most prevalent RNA modifications, first characterised as 5‐ribosyluracil in 1959. Due to its abundance, it has been referred to as the ‘fifth ribonucleoside’ and later renamed pseudouridine.^2^, ^3^, ^4^, ^5^ Ψ is found in nearly all RNA types, playing a crucial role in protein synthesis. Pseudouridylation, an enzymatic isomerisation that converts uridine (U) to Ψ in RNA molecules,^6^ accounts for.2–.6% of U in mammalian mRNAs^7^ and is mediated by two distinct catalytic mechanisms. This modification is dynamic, reversible, and inducible.^8^ In contrast to the highly abundant N^6^‐methyladenosine (m^6^A), which exhibits positional preference near long internal and external exons, termination codons, and the 3′ untranslated region (3′‐UTR), pseudouridylation occurs without positional bias in the 3′‐UTR, coding sequences (CDS), and 5′ untranslated region (5′‐UTR) of mRNA.^9^, ^10^ Imbalances in pseudouridylation contribute to pathogenesis by altering RNA fate, affecting processes such as pre‐mRNA splicing, mRNA stability, tRNA transport, mRNA‐tRNA interactions, and ribosomal fidelity. These processes regulate numerous biological functions, including gene expression and signal transduction. Dysregulated pseudouridylation is commonly observed in various cancers, including colorectal cancer (CRC),^11^ ovarian cancer,^12^ and glioblastoma,^13^ accelerating tumour proliferation, invasion, and metastasis. Additionally, similar pseudouridylation imbalances have been implicated in non‐cancerous diseases, such as dyskeratosis congenita, neurological disorders, and metabolic diseases.^14^, ^15^ Intriguingly, aberrant pseudouridylation can be induced by cellular stresses, including heat shock and nutrient deprivation, potentially contributing to disease progression.^8^, ^16^ As such, targeting pseudouridylation presents a promising therapeutic avenue, supported by advances in drug discovery and design. This review offers a comprehensive analysis of pseudouridylation‐mediated biological processes, the relevance to disease, the external stresses that trigger this modification, and the therapeutic potential in treating human diseases.

## 2 METHODS TO DETECT PSEUDOURIDINE (Ψ) AND CURRENT RESEARCH PROGRESS

Precise and sensitive detection of pseudouridine abundance and distribution is critical for advancing RNA pseudouridylation research. However, due to the identical molecular weight and similar base‐pairing characteristics between pseudouridine and uridine during reverse transcription, detecting pseudouridine residues experimentally becomes increasingly challenging. Additionally, variations in site detection results stemming from different methods complicate the study of pseudouridylation‐related diseases. A range of detection techniques has been developed, from classic mass spectrometry‐based methods to newer approaches such as nanopore‐based technologies and BID sequencing, each with its advantages and limitations.

### 2.1 Classic mass spectrometry‐based methods

Mass spectrometry is commonly used to detect pseudouridine modifications in RNA. Recently, mass spectrometry‐based techniques have been introduced to map pseudouridine sequences in RNA. However, mass spectrometry primarily provides qualitative data regarding the presence or absence of pseudouridine in RNA molecules. To enable both qualitative and quantitative analysis of pseudouridine, liquid chromatography‐tandem mass spectrometry (LC‐MS/MS) methods have been introduced,^17^ though these remain limited to purified RNA species.

### 2.2 High‐throughput sequencing‐based approaches

Historically, the most widely employed technique for detecting pseudouridine involved treatment with N‐cyclohexyl‐N‐β‐(4‐methylmorpholinium) ethylcarbodiimide p‐tosylate (CMCT). CMCT reacts with Ψ to form a stable adduct, which inhibits reverse transcriptase and results in truncated cDNA. This method has been applied in polyacrylamide gel electrophoresis and autoradiography for detecting pseudouridine in abundant RNA species, such as rRNA.^18^ High‐throughput RNA sequencing methods combined with CMCT treatment have also been employed to detect pseudouridine in low‐abundance transcripts. In addition, emerging sequencing technologies, including Nanopore sequencing and BID sequencing, have been developed to map pseudouridylation profiles.

Nanopore sequencing enables detection of known pseudouridine sites (Ψ sites) in mRNA, ncRNA, and rRNA, and can identify novel sites as well. By integrating the nanoRMS algorithm^19^ with the nanoCMC sequencing method—based on traditional CMC processing techniques—this approach enhances the accuracy of pseudouridylation site identification. Although Nanopore sequencing offers advantages such as extremely long read lengths, high real‐time performance, and low cost, challenges remain, including a high sequencing error rate that necessitates algorithmic corrections. Further improvements in spatial resolution and sequencing throughput are needed. Moreover, Nanopore sequencing is dependent on optimised base‐calling algorithms for detecting modifications, and its accuracy may be influenced by sequencing errors and algorithmic limitations.

The BID sequencing method (BID seq), a deletion sequencing technique induced by bisulphite (BS), enables high‐throughput detection and precise single‐base localisation of pseudouridylation. This method quantitatively converts Ψ into ‘Ψ‐BS adducts’, resulting in base deletion signals at Ψ‐containing sites following reverse transcription, thereby facilitating quantitative sequencing of Ψ modifications in RNA at single‐base resolution. BID seq has been successfully employed for quantitative sequencing, revealing high levels of pseudouridine in mammalian mRNA at single‐base resolution., and confirming the role of Ψ in facilitating stop codon readthrough in vivo.^20^ This technique offers high sensitivity and specificity for detecting Ψ modifications in mRNA at single‐base resolution, making it valuable for studying their biological functions. However, it requires specific chemical reaction conditions and sequencing techniques, which may pose technical challenges and increase costs.

Additionally, a novel fluorescence‐based quantitative PCR method has been developed for detecting pseudouridine.^21^ This approach is simple, fast, and avoids the use of radioactive materials, offering a straightforward detection process for targeted pseudouridine. However, its sensitivity may be limited by the efficiency and specificity of PCR amplification, making it less effective for detecting low‐abundance pseudouridylation sites.

**FIGURE 1:**
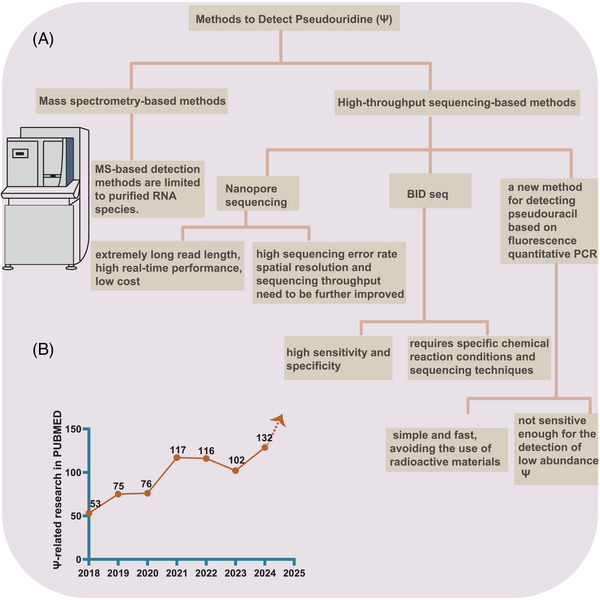
Detection methods for pseudouridine (Ψ) and current research advances. (A) Overview of various pseudouridine detection techniques, ranging from classic mass spectrometry‐based methods to newer approaches such as nanopore‐based technologies and BID sequencing. The advantages and limitations of each method are discussed. (B) The volume of literature on pseudouridylation modification research has steadily increased, further underscoring its importance in disease studies.

In summary, while various methods for detecting pseudouridylation offer distinct advantages, they also come with limitations. The variability in detected sites across different technologies remains a significant challenge in pseudouridylation research, particularly in understanding pseudouridine‐related diseases. Future advancements should concentrate on ameliorating the accuracy and consistency of detection methods (Figure 1A).

Notably, recent computational efforts have made significant strides in this area. For instance, two bioinformatics databases, RMDisease and RMVar, have predicted numerous disease‐related Ψ loci in humans, providing valuable insights for related research. RMDisease v2.0 (http://www.rnamd.org/rmdisease2/) reports an aggregate of 1 366 252 RNA modification‐related variants, which may impact modification sites, involving N6 methyladenosine (m^6^A), 5‐methylcytosine (m^5^C), N1 methyladenosine (m^1^A), 5‐methyluridine (m^5^U), pseudouridine (PSI), N6, 2′‐O‐dimethyladenosine (m^6^Am), N7 methylguanosine (m^7^G), adenosine‐to‐inosine (a‐to‐I), N4‐acetylcytidine (ac4C), and 2′‐O‐methylation (Am), etc. These variants span 20 species, including humans, mice, rats, zebrafish, corn, fruit flies, and yeast.^22^, ^23^ RMVar 2.0 (https://rmvar.renlab.cn/#/home) is an updated database developed by a research team from Sun Yat‐sen University, focusing on functional variants associated with RNA modifications. This tool aims to deepen understanding of the relationship between genetic variants and RNA modification.^24^

Due to past limitations in detection technologies, despite recognising the significant impact of pseudouridylation on human diseases, its mechanistic effects remain poorly understood. However, with the rapid development of emerging detection technologies, the volume of literature on pseudouridylation modification research has steadily increased, further underscoring its importance in disease studies (Figure 1B). Research on pseudouridylation has largely concentrated on the pathogenesis and therapeutic targeting of tumours, with increasing attention also being paid to non‐tumour diseases. The growing number of research, as evidenced by trends on PubMed, reflects a shift toward high‐quality studies in this field.

## 3 PSEUDOURIDYLATION PROCESS AND ITS BIOLOGICAL SIGNIFICANCE

**FIGURE 2:**
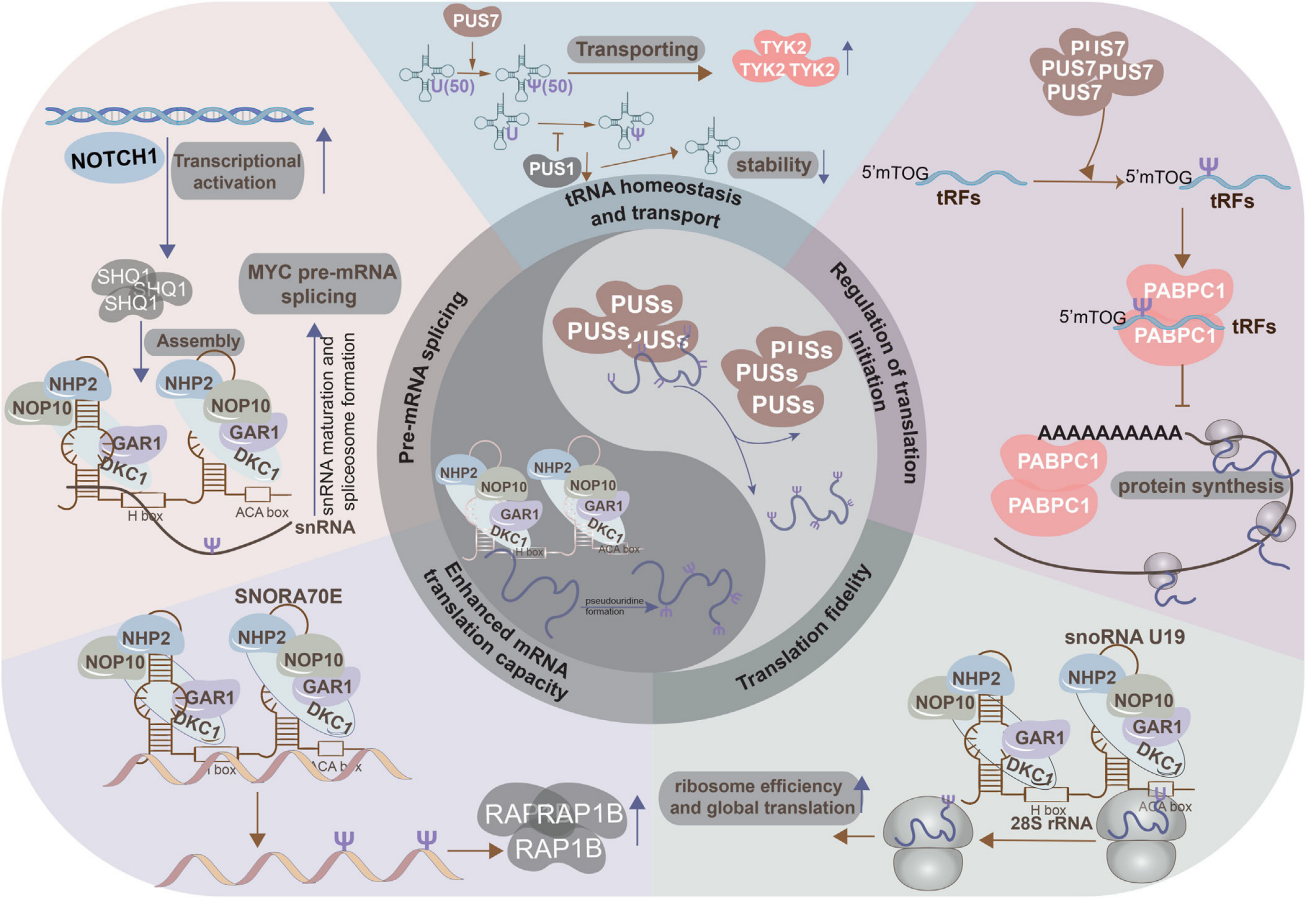
Pseudouridylation process and biological significance manipulated by pseudouridylation in diseases. Pseudouridylation, catalysed by pseudouridine synthases (PUSs), follows two primary pathways: RNA‐independent and snoRNA‐dependent pseudouridylation. RNA pseudouridylation is crucial for various biological processes, including tRNA homeostasis, tRNA transport, translation initiation regulation, pre‐mRNA splicing, enhancement of mRNA translation, and translational fidelity.

Pseudouridylation is catalysed by pseudouridine synthases (PUSs) via two distinct pathways: RNA‐independent pseudouridylation and snoRNA‐dependent pseudouridylation.^6^, ^13^, ^25^, ^26^ The RNA‐independent pathway, also known as the protein‐only mechanism, is driven by a single PUS enzyme that independently identifies and modifies RNA substrates.^6^ PUS enzymes are categorised into six different families: TruA, TruB, TruD, RsuA, RluA, and Pus10.^13^, ^27^, ^28^ The human homologs of these enzymes include RNA pseudouridine synthases (RPUSD1‐4), PUS1‐4, PUS6, PUS7, PUS7L, PUS9, and PUS10.^29^, ^30^ Despite low sequence identity, PUSs in the RNA‐independent mechanism share a conserved structural fold, consisting of an eight‐stranded mixed β‐sheet, flanked by helices and loops surrounding the active site.^27^, ^31^ In contrast, snoRNA‐dependent pseudouridylation relies on a complex involving a Box H/ACA RNA, which adopts a characteristic hairpin‐hinge‐hairpin‐tail structure, along with four core proteins: centromere‐binding factor 5 (Cbf5, also known as dyskerin in mammals), glycine‐arginine‐rich protein 1 (Gar1), non‐histone protein 2 (Nhp2), and nucleolar protein 10 (Nop10).^27^, ^31^ Within this complex, Cbf5 (dyskerin) serves as the PUS enzyme. The RNA substrate is recognised upon entering the pseudouridylation pocket formed by Box H/ACA RNA. It then binds to the RNA‐protein complex through complementary base pairing, enabling Cbf5 (dyskerin) to catalyse the pseudouridylation of the RNA^6^ (Figure 2, Table 1). Altogether, evaluating the two pseudouridylation pathways—RNA‐independent by PUS enzymes or via the snoRNA‐protein complex—would offer valuable insights.

**TABLE 1:** Molecules and molecular characteristics.

Gene name	Enzyme	Catalytic domain family	Molecular weights	Cellular location	Substrate
PUS1 (MLASA1)	Pseudouridine synthase 1 tRNA pseudouridine synthase A	TruA	47.47 kDa	Nucleus, cytoplasm	tRNA, snRNA, mRNA
TRUB2	TruB pseudouridine synthase 2	TruB	36.69 kDa	Mitochondrion matrix	tRNA
PUS3	Pseudouridine synthase 3	TruA	55.65 kDa	Nucleus	tRNA, mRNA
PUS4 (TruB1)		TruB	37.25 kDa	Nucleus, cytoplasm	mRNA
PUS5	Pseudouridine synthase 5	RluA			rRNA
RPUSD1	RNA Pseudouridine Synthase Domain Containing 1	RluA	34.76 kDa	unknown	Unknown
RPUSD2	RNA Pseudouridine Synthase Domain Containing 2	RluA	61.31 kDa	Unknown	Unknown
RPUSD3	RNA Pseudouridine Synthase Domain Containing 3	RluA	38.46 kDa	Mitochondrion matrix,	rRNA
RPUSD4	RNA Pseudouridine Synthase Domain Containing 4	RluA	42.21 kDa	Mitochondrion matrix, Nucleus, cytoplasm	rRNA
PUS7	Pseudouridine synthase 7	TruD	75.03 kDa	Nucleus	tRNA, mRNA
PUS7L	Pseudouridine synthase 7 homolog‐like protein	TruD	80.70 kDa	Nucleus	tRNA
PUS10	Pseudouridine synthase 10		60.24 kDa	Mitochondrion, Nucleus, cytoplasm	tRNA
DKC1	Dyskerin	TruB	57.67 kDa	Nucleus, cytoplasm	rRNA, sno/scaRNA, snRNA

In cellular processes, RNA pseudouridylation exerts a critical effect on the metabolism of various RNA types, particularly tRNA, rRNA, snoRNA, and mRNA. This RNA decoration contributes to the complexity of the cellular proteome by regulating protein synthesis. This study focuses on the biological significance of pseudouridylation across different RNA types, highlighting its role in tRNA homeostasis and transport, regulation of translation initiation, pre‐mRNA splicing, enhancement of mRNA translation capacity, and maintenance of translational fidelity.

### 3.1 tRNA homeostasis and transport

For a long time, research on tRNAs, including pseudouridine profiling, was limited due to the complex secondary structure of tRNA.^4^, ^5^, ^7^, ^32^, ^33^ However, advancements in high‐throughput sequencing technologies, such as DM‐tRNA‐seq, which enables annotation of the majority of tRNAs for six specific base modifications, have gradually revealed the modification landscape of tRNA.^34^, ^35^, ^36^, ^37^, ^38^ Of note, increasing evidence demonstrates that tRNA pseudouridylation plays a critical role in both physiological and pathological states by influencing tRNA homeostasis and transport. The most frequently identified pseudouridylation site is Ψ55 in the TΨC loop, followed by Ψ27, Ψ28, Ψ38, and Ψ39 in the anticodon stem‐loop region. Pseudouridylation at other sites, including Ψ50, Ψ54, Ψ35, and Ψ36, has also been observed to varying extents,^37^, ^39^ determining the tRNA transport function and fate. For instance, PUS7 facilitates pseudouridylation at Ψ50 in several tRNA isoforms, involving tRNA‐Arg‐CCG, tRNA‐Gln‐CTG, tRNA‐Asp‐GTC, tRNA‐Glu‐CTC, and tRNA‐Tyr‐GTA, enhancing the translation of tyrosine kinase 2 (TYK2) by mediating the transport of specific amino acids in glioblastoma.^13^ Furthermore, PUS1 downregulation induced by P175fs mutation reduces the mitochondrial protein synthesis via pseudouridylating mitochondrial tRNAs involving mt‐tRNA^Cys^, mt‐tRNA^Ser (UCN)^, and mt‐tRNA^Tyr^ at Ψ28, contributing to mitochondrial myopathy, lactic acidosis, and sideroblastic anaemia syndrome (MLASA).^40^ Additionally, PUS10 exerts a physiological effect on governing human cell growth via catalysing the pseudouridylation of cytoplasmic tRNAs at Ψ54.^37^ Overall, tRNA pseudouridylation is essential for maintaining tRNA transport and cellular protein synthesis. Given its profound biological function, further research into the role of tRNA pseudouridylation is imperative.

### 3.2 Regulation of translation initiation

Translation occurs in four primary stages: initiation, elongation, termination, and ribosome recycling. Among these, initiation serves as the pivotal control point, where the ribosome is positioned at the start codon with an initiator tRNA, primed to begin elongating the protein chain.^41^ Recent research highlights the critical role of pseudouridylation in the initiation phase of translation. Concretely, PUS7‐mediated pseudouridylation significantly enhances the interaction between mini transfer RNA‐derived fragments (tRFs) containing a 5′ terminal oligoguanine (mTOG) and poly(A)‐binding protein cytoplasmic 1 (PABPC1), which associates with eukaryotic translation initiation factor 4 gamma (eIF4G) to facilitate the formation of the eIF4F complex. This interaction inhibits translation and exerts a vital effect on the translational control of stem cells.^42^, ^43^ In myelodysplastic syndrome, PUS7‐mediated pseudouridylation of mTOG promotes a direct Ψ‐dependent interaction with PABPC1, disrupting the recruitment of the translational co‐activator PABPC1‐interacting protein 1 (PAIP1)^44^, ^45^ significantly hindering the translation of transcripts with pyrimidine‐rich sequences (PES) in the 5′‐UTR, such as 5′ terminal oligopyrimidine (TOP) tracts, which encode components of the protein synthesis machinery and are frequently remodelled in tumours.^46^

Pseudouridylation functions as a structural ‘switch’, facilitating conformational changes that modulate the interactions between mTOG RNA and mTOG‐associated proteins, which are critical for eIF4F assembly. Thus, pseudouridylation plays an essential role in regulating the initiation phase of protein translation.

### 3.3 Pre‐mRNA splicing

In eukaryotes, intron excision from pre‐mRNA by the spliceosome is a critical step in gene expression regulation, with small nuclear (sn) RNAs (U1, U2, U4, U5, and U6) playing essential roles. These snRNAs, in conjunction with multiple proteins, form dynamic small nuclear ribonucleoprotein (snRNP) complexes, which are integral to the spliceosome's structure.^47^ Among the post‐transcriptional modifications in all five types of snRNAs, pseudouridylation is the most significant. Mammalian snRNAs carry 27 pseudouridylation,^48^, ^49^ with snRNA U2 being the most extensively modified, featuring 14 Ψs, including the conserved Ψ34, Ψ41, and Ψ43 across species.^33^ Recent research has shown that these modified residues contribute to snRNP and spliceosome assembly in the splicing process. Pseudouridylation sites on snRNAs are primarily located in regions crucial for RNA‐RNA and protein‐RNA interactions, emphasising their importance in the multistep pre‐mRNA splicing process. In leukaemia, SHQ1, a factor for H/ACA snoRNP assembly, facilitates U2 snRNA pseudouridylation at Ψ54, therefore, promoting the proto‐oncogene *MYC* pre‐mRNA splicing and tumour cell survival.^50^ In glioblastoma, miR‐10b enhances the pseudouridylation of snRNA U6 at position 86 to increase its stability and binding affinity to splicing factors spliceosome‐associated factor 3 (SART3) and pre‐mRNA processing factor 8 (PRPF8), contributing to global splicing alterations and tumourigenesis.^51^ Additionally, during embryonic development, the pseudouridylation level of snRNA U2 is regulated by small Cajal body‐specific RNA (scaRNA), ensuring spliceosome fidelity and enabling selective mRNA splicing.^52^ In addition to m^6^A‐mediated splicing regulation, pre‐mRNA pseudouridylation is likely to reveal a new mechanism for the nuclear splicing.

### 3.4 Enhanced mRNA translation capacity

The conversion of mRNA into protein and its subsequent folding into an active form are fundamental to nearly all cellular processes, representing the cell's most substantial energy expenditure.^53^ To date, merely three enzymes have been demonstrated to induce mRNA pseudouridylation in human cells, including PUS1, PUS7 and TRUB1. Among them, PUS1 and PUS7‐mediated pseudouridylation has been demonstrated to enhance mRNA translation efficiency. For example, PUS1 boosts the translation capacity of several oncogenic mRNAs, such as insulin receptor substrate 1 (*IRS1*), serine hydroxymethyltransferase 2 (*SHMT2*), *MDM2* and *c‐MYC*, in a pseudouridylation‐dependent manner, thereby promoting tumourigenesis in hepatocellular carcinoma (HCC).^54^ Of note, nanopore sequencing has identified Ψ sites with varying stoichiometries, revealing 8624 PUS7‐dependent modifications across 1246 mRNAs. These mRNAs predominantly encode proteins involved in ribosome assembly, translation, and energy metabolism.^55^ Recent studies show that PUS7 pseudouridylates Alpha‐ketoglutarate‐dependent Dioxygenase alkB Homolog 3 (*ALKBH3*) mRNA by modifying the U696 site, enhancing its translation efficiency and suppressing gastric tumourigenesis, with correlated expression serving as potential prognostic biomarkers and therapeutic targets in gastric cancer.^56^ Pseudouridylation‐induced enhancement of mRNA translation capacity has profound significance in the field of biomedicine, such as mRNA vaccine development, gene therapy, and regenerative medicine, providing strong support for the widespread application of mRNA technology.

### 3.5 Translational fidelity

Translating genetic information into functional proteins is a complex, multistep process, with each stage meticulously regulated to ensure translational accuracy and maintain cellular health.^57^ Among the various post‐transcriptional modifications, pseudouridylation plays a critical role in human ribosomes,^58^ manipulating the translation efficiency and accuracy via regulating the interactions between ribosomes and tRNA, mRNA, or translation factors.^59^, ^60^, ^61^ Consequently, pseudouridylation in rRNA significantly impact both the speed and fidelity of translation.^61^, ^62^, ^63^ For example, pseudouridylation at positions Ψ4331 and Ψ496 is markedly decreased in ribosomes, disruption of pseudouridylation impairs internal ribosome entry site (IRES)‐dependent RNA translation, compromising translational accuracy and resulting in protein synthesis irregularities that undermine hematopoietic stem cell (HSC) function in patients with familial X‐linked dyskeratosis congenita (DC).^61^, ^64^, ^65^, ^66^ Additionally, snoRNA U19, which mediates the two most conserved pseudouridylation modifications on 28S ribosomal RNA (rRNA), is essential for maintaining ribosome function. Overexpression of snoRNA U19 enhances pseudouridylation of 28S rRNA, improving ribosomal efficiency and global translation.^33^, ^67^ Furthermore, the absence of dyskerin pseudouridine synthase 1 (DKC1), an enzyme responsible for rRNA pseudouridylation, leads to defects in translation initiation and fidelity in mammalian cells.^68^ Although the precise mechanism remains unclear, it is thought to involve pseudouridine‐induced changes in RNA conformation.^69^ Dysregulation of rRNA pseudouridylation primarily affects translational fidelity by altering rRNA structure, which in turn governs numerous biological processes. Given the crucial role of rRNA in ribosome function and its involvement in diverse physiological and pathological processes through RNA epigenetic modifications, further investigation into the role of pseudouridylation in rRNA metabolism is essential.

Pseudouridylation of mRNA is also predicted to play a crucial role in maintaining translational fidelity via manipulating the decoding of both nonsense and sense codons. For example, artificial pseudouridylation of stop codons has been demonstrated to induce over 70% stop codon readthrough in rabbit reticulocyte lysate,^70^ with similar efficiency observed in E. coli lysate.^71^ However, in human HEK293T cells, pseudouridylated stop codons embedded in synthetic mRNAs, where all uridine residues are substituted with Ψ, appear to terminate translation correctly.^72^ Notably, the nonsense suppression effect mediated by Ψ has thus far only been observed in engineered systems, and the readthrough of endogenous Ψ‐modified termination codons in vivo has yet to be characterised.^33^ Similarly, pseudouridylation also modulates the decoding of sense codons, although research findings have been inconsistent. While some studies show that entirely pseudouridylated mRNAs transfected into human cells generate functional proteins,^72^, ^73^, ^74^ one study reports Ψ‐dependent mistranslation of peptides,^73^ suggesting that pseudouridylation can affect the decoding of both sense and nonsense codons, thereby influencing translational fidelity. As transcriptome‐wide pseudouridine sequencing technologies advance, a growing number of pseudouridylation sites within mRNAs are being identified in human cells,^5^, ^75^, ^76^ shedding light on its true role in health and disease.

The impact of pseudouridylation on translational fidelity is primarily mediated through modifications in rRNA and mRNA codons. Pseudouridylation of rRNA, an essential component of ribosomes, alters translational fidelity by modulating interactions between ribosomes, tRNA, and mRNA. Additionally, pseudouridylation of mRNA codons impairs the accurate recognition and translation of these codons, thus disturbing the fidelity of protein synthesis.

## 4 STIMULATION OF RNA PSEUDOURIDYLATION BY CELLULAR STRESSES

**FIGURE 3:**
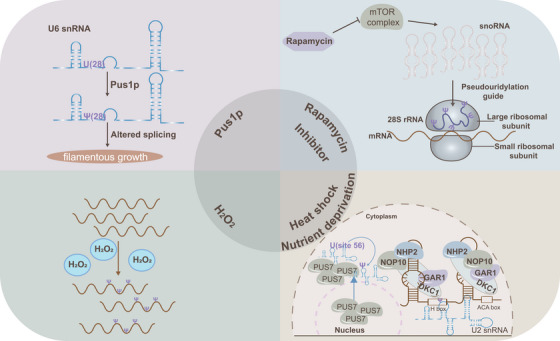
Dysregulation of pseudouridylation in human diseases. Pseudouridylation is implicated in the acquisition and maintenance of cancer hallmarks, such as tumour invasion, metastasis, cancer stem cell self‐renewal, immune evasion, and resistance to programmed cell death. Conversely, a reduction in pseudouridylation levels is primarily associated with non‐tumour diseases.

Mechanism research reveals that pseudouridylation plays a vital role in both physiological and pathological states. Notably, recent studies have revealed that it can be dynamically induced under cellular stress conditions such as heat shock and nutrient deprivation. For example, in *Saccharomyces cerevisiae*, Pus7p, which typically catalyses pseudouridylation at position 35 of snRNA *U2*, also facilitates pseudouridylation at position 56 under conditions of nutrient deprivation or heat shock. Similarly, the wild‐type snR81 RNP, responsible for pseudouridylation at position 42 of snRNA *U2* via the 5′ pocket and at position 1051 of 25S rRNA via the 3′ pocket, can also induce pseudouridylation at position 93 (via the 3′ pocket) during nutrient deprivation.^8^, ^16^ The functional significance of inducible pseudouridylation is also notable. For instance, pseudouridylation of snRNA *U2* at Ψ93 has been shown to reduce pre‐mRNA splicing efficiency.^8^ Additionally, during yeast filamentous growth, snRNA *U6* undergoes pseudouridylation at U28 by Pus1, an RNA‐independent pseudouridine synthase. This modification is critical, as the H/ACA RNA box activates filamentous growth by targeting pseudouridylation at U28 in snRNA *U6*, and preventing U6‐Ψ28 formation impedes this growth.^77^ Beyond heat shock and nutrient deprivation, oxidative stress induced by H_2_O_2_ treatment has been shown to alter mRNA pseudouridylation levels in HEK293T cells.^7^ Furthermore, repression of the mTOR pathway has been shown to slightly increase pseudouridylation levels in rRNA of Chinese hamster ovary cells, although the specific sites remain unidentified.^67^ Heat shock can also translocate yeast nuclear Pus7 to the cytoplasm, leading to pseudouridylation of cytoplasmic substrates.^76^ These findings suggest that cellular stresses may induce changes in pseudouridylation levels through the generation and altered localisation of PUSs, the ‘executors’ of this modification. The disruption of cellular homeostasis caused by these stresses may impact disease development across various cell types, further supporting pseudouridylation's involvement in disease mechanisms (Figure 3).

Heat shock and nutrient deprivation are currently the primary cellular stresses known to induce pseudouridylation with the underlying mechanisms remaining enigmas. However, these findings hint that pseudouridylation plays a vital role in responding to cell stress to maintain cell vitality. Diseases often induce the changes of cellular internal and external environment, such as metabolic reprogramming, oxidative stress, inflammatory response, extracellular matrix reconstruction, pathogen infection, and hypoxia. In turn, pseudouridylation as a dynamic and complex process is regulated by diverse internal and external stress to adapt cellular function requirements or respond to environmental alteration, indicating that pseudouridylation may participate in the development of diseases.

## 5 DYSREGULATION OF PSEUDOURIDYLATION IN HUMAN DISEASES

**FIGURE 4:**
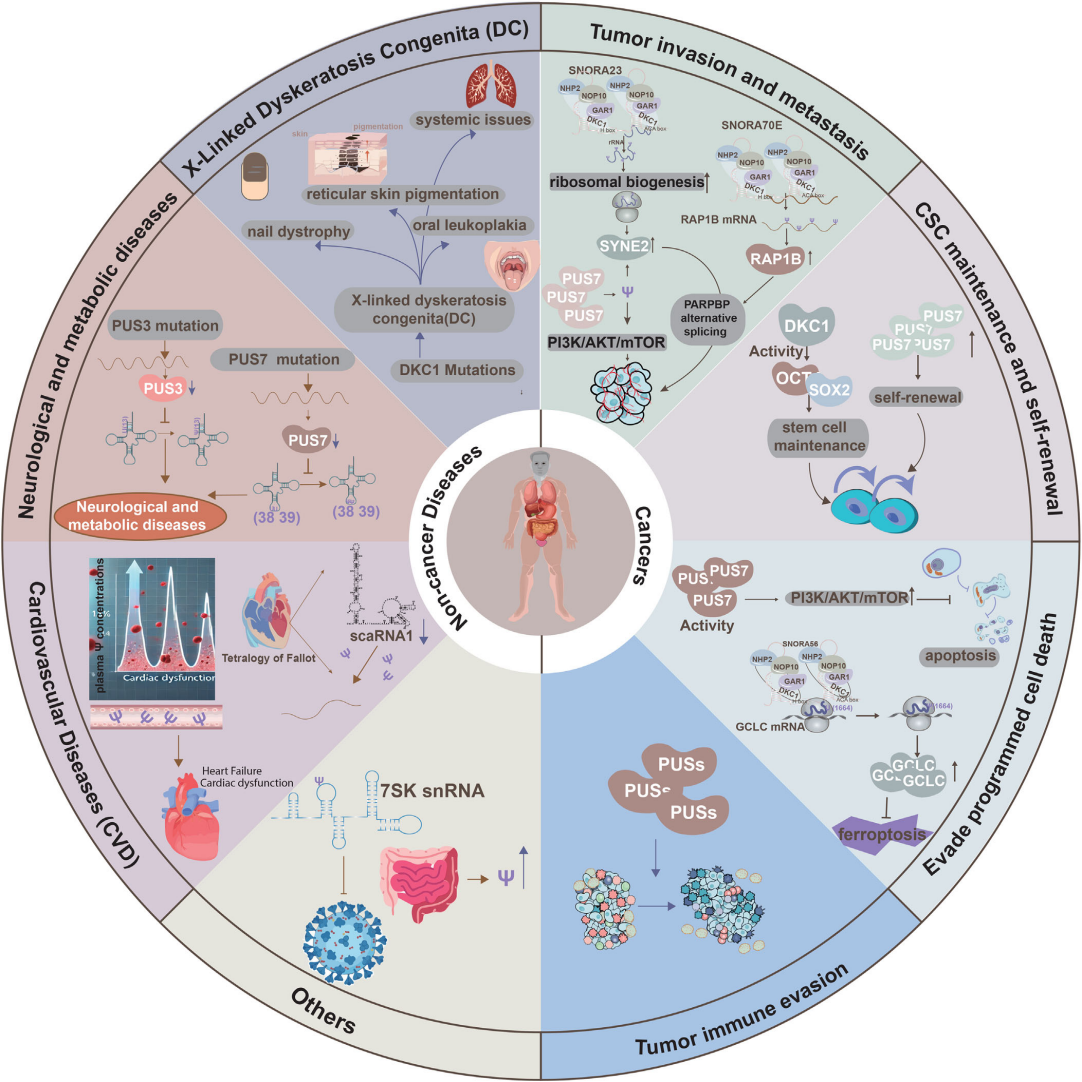
Cellular stressors inducing pseudouridylation in diseases. Pseudouridylation plays a critical role in cellular homeostasis and is triggered by various stressors, including heat shock and nutrient deprivation, the primary known inducers of this modification. Additionally, oxidative stress induced by H_2_O_2_ has been shown to alter mRNA pseudouridine levels in HEK293T cells. Repression of the mammalian target of the rapamycin (mTOR) pathway has also been linked to increased pseudouridine levels in rRNA, though the exact sites remain unidentified.

Growing evidence establishes a strong link between RNA epigenetic modifications and human diseases, with pseudouridylation emerging as a critical factor in both tumourigenesis and non‐tumour pathologies. Increased pseudouridylation is frequently associated with tumour initiation, progression, and poor prognosis, whereas its reduction is predominantly implicated in non‐tumour diseases. This review focuses on the dysregulation of pseudouridylation in human diseases, aiming to enhance the understanding of the molecular mechanisms that connect RNA epigenomics to pathogenesis (Figure 4, Table 2).

**TABLE 2:** Function and mechanism of pseudouridylation molecules in diseases.

Disease	PUS(s)/snoRNA involved	Molecular effect	Function and mechanism	References
Cancer				
Pancreatic ductal adenocarcinoma (PDAC)	SNORA23	Upregulated	Promote tumourigenicity and metastasis via enhancing the expression of spectrin repeat containing nuclear envelope protein 2 (SYNE2) by regulating ribosomal biogenesis	^78^
Ovarian cancer (OC)	SNORA70E	Upregulated	Enhance cell proliferation, invasion, and migration in vitro, facilitating tumour progression via the pseudouridylation of RAP1B and alternative splicing of PARPBP	^12^
Colon cancer	PUS7	Upregulated	Promote malignant phenotypes by triggering the PI3K/AKT/mTOR signalling pathway	^83^
Glioblastoma (GBM)	PUS7	Upregulated	Facilitate pseudouridylation at position 50 in several tRNA isoforms, thereby regulating the translation of tyrosine kinase 2 (TYK2) by transporting specific amino acids to promote tumourigenicity and self‐renewal capacity and frequency	^13^
Non‐small cell lung cancer (NSCLC)	DKC1	Upregulated	LncRNA PCAT1 interacts with DKC1 to regulate proliferation, invasion, and apoptosis via the VEGF/AKT/Bcl2/Caspase9 pathway	^136^
Myelodysplastic syndromes (MDS)/ Acute myeloid leukaemia (AML)	PUS7	Deletion	PUS7 inactivation in embryonic stem cells impairs tRF‐mediated translation regulation, leading to increased protein biosynthesis and defective germ layer specification	^42^
Hepatocellular carcinoma (HCC)	PUS1	Upregulated	Facilitate the pseudouridylation of mRNA, thereby increasing the protein expression levels of several oncogenes, including insulin receptor substrate 1 (IRS1) and c‐MYC, thereby promoting tumourigenesis	^54^
Non‐cancer human diseases				
X‐linked dyskeratosis congenita (DC)	DKC1	Recessive mutation	Disrupted pseudouridylation influences the internal ribosome entry site (IRES)‐dependent RNA translation, compromising the accuracy of translation and resulting in irregularities in protein synthesis that can undermine the function of hematopoietic stem cells	^76^, ^97^
MLASA (Mitochondrial Myopathy and Sideroblastic Anaemia)	PUS1	Missense mutation	Oxidative phosphorylation provoked by PUS1 gene missense mutations, resulting in the partial or complete loss of pseudouridylation at specific tRNA sites	^104^
Neurological and metabolic diseases	PUS3, PUS7	Missense/frameshift/ truncation mutations	Reduced PUS7 mRNA expression in patient fibroblasts, leading to decreased pseudouridylation at position 13 of tRNAs, a novel homozygous truncating mutation in PUS3, leading to reduced pseudouridylation at positions 38 and 39 in tRNAs	^101^, ^102^, ^103^

### 5.1 Cancers

#### 5.1.1 Pseudouridylation‐mediated tumour invasion and dissemination

The invasive‐metastatic cascade, a defining feature of tumour cell dissemination, represents the most lethal aspect of cancer progression. Substantial evidence highlights the role of pseudouridylation, mediated by snoRNAs and their associated enzymes, in promoting tumour invasion and dissemination across various cancer types. In pancreatic ductal adenocarcinoma (PDAC), increased pseudouridylation of rRNA, facilitated by snoRNA *SNORA23*, correlates with enhanced tumour invasion and poorer patient survival. Mechanistically, *SNORA23* may regulate ribosomal biogenesis and upregulate spectrin repeat‐containing nuclear envelope protein 2 (SYNE2), thereby supporting PDAC cell survival, invasion, metastasis, and tumour growth in mouse xenograft models.^78^ Although direct evidence linking *SNORA23* to invasion and metastasis via pseudouridylation is limited, its role in mediating pseudouridylation indirectly supports this association. Despite frequent alterations in snoRNA expression in tumours, few studies have directly linked these changes to corresponding pseudouridylation modifications. However, emerging research has associated abnormal snoRNA expression with processes such as apoptosis inhibition, uncontrolled cell proliferation, angiogenesis, and metastasis.^79^ Hence, snoRNAs may exert secondary effects that are dependent on pseudouridylation, further promoting cancer progression.^80^, ^81^ Notably, growing evidence has revealed the relationship between snoRNA expression and pseudouridylation. In ovarian and colon cancers, overexpression of SNORA70E and PUS7, respectively, has been demonstrated to enhance cell propagation, invasion, and migration, thereby facilitating tumour progression. Specifically, overexpression of *SNORA70E* enhances tumour cell propagation, invasion, and migration in vitro, driving tumour progression via pseudouridylation of *RAP1B* and alternative splicing of *PARPBP* in ovarian cancer.^12^ Similarly, in high‐grade ovarian cancer, elevated snoRNA expression correlates with increased rRNA modifications, primarily pseudouridylation, which contributes to tumour migration.^82^ PUS7 also promotes malignant phenotypes in colon cancer by stimulating the PI3K/AKT/mTOR signalling pathway.^83^ While the precise role of PUS7 as a pseudouridylate synthase in promoting colon cancer metastasis remains unclear, current evidence merely suggests that pseudouridylation plays a significant role in promoting tumour malignant phenotypes. In non‐small cell lung cancer (NSCLC), increased pseudouridylation, mediated by specific H/ACA box snoRNAs and NOP10 protein, has been identified. Elevated pseudouridylation at 10 H/ACA box snoRNA target sites on rRNA is observed in tumour samples, with loss of NOP10 leading to reduced levels of H/ACA box snoRNAs and rRNA pseudouridylation, which in turn impairs lung cancer migration and invasion.^84^ Collectively, these findings underscore the critical role of pseudouridylation, mediated by diverse snoRNA‐induced modifications, in driving the invasive and metastatic potential of various cancer types.

#### 5.1.2 Pseudouridylation‐maintained cancer stem cell self‐renewal

Cancer stem cells (CSCs), with their potent self‐renewal and tumourigenic capabilities, are critical drivers of tumour progression and therapy resistance. Recent studies illustrate that pseudouridylation may play a key role in CSC maintenance, potentially influencing therapeutic outcomes. Pseudouridylation, a modification that alters RNA structure and function—vital for translation, gene regulation, and telomere maintenance—has been implicated in stem cell regulation.^85^ Mutations in dyskerin, the enzyme that catalyses RNA‐dependent pseudouridylation, disrupt stem cell functions in both murine and human models.^85^ For instance, dyskerin mutations impair HSC differentiation,^86^ while DKC1 deficiency in adult mice results in early embryonic lethality and age‐related depletion of HSCs, driven by DNA damage and growth defects.^87^ Dyskerin also regulates pluripotency factors such as organic cation/carnitine transporter4 (OCT4) and SRY‐box transcription factor 2 (SOX2), suggesting that its expression levels may influence stem cell pluripotency.^88^ In glioblastoma, elevated PUS7 expression correlates with poor prognosis, and PUS7 knockout in glioblastoma stem cells (GSCs) significantly reduces their self‐renewal capacity and frequency.^13^ Likewise, PUS10 expression increases in aged hematopoietic stem and progenitor cells due to reduced CRL4DCAF1‐mediated ubiquitination, driving aging phenotypes in HSCs.^89^ These findings highlight that pseudouridylation is crucial for maintaining CSC properties, presenting potential therapeutic targets.

#### 5.1.3 Pseudouridylation‐involved programmed cell death escape

In cancer cells, pseudouridylation disrupts programmed cell death (PCD) pathways, promoting tumour cell survival and resistance to treatment. Pseudouridylation contributes to the dysregulation of apoptosis, a key PCD process that eliminates dysfunctional and aberrant cells.^90^ PUS7, a pseudouridine synthase, modulates pseudouridylation levels in tumour cells and influences the Akt kinase (AKT) signalling pathway, which suppresses apoptosis. For instance, PUS7 overexpression enhances proliferation, invasion, and resistance to apoptosis via the phosphatidylinositol 3‐kinase (PI3K)/AKT/mechanistic target of rapamycin kinase (mTOR) pathway in colon cancer cells.^83^ Furthermore, PUS10 translocates to the mitochondria during TNF superfamily member 10 (TRAIL)‐induced apoptosis, forming a caspase‐3 amplification loop that disrupts apoptosis and promotes tumour progression.^91^ Pseudouridylation also plays a role in cancer‐associated ferroptosis. *SNORA56*‐mediated pseudouridylation of 28S rRNA inhibits ferroptosis and promotes CRC growth by enhancing glutamate cysteine ligase (GCLC) translation. Specifically, *SNORA56* directs pseudouridylation at the U1664 site of 28S rRNA, boosting the translation of the catalytic subunit of GCLC and preventing cellular peroxide accumulation.^11^ These insights into the role of pseudouridylation in apoptosis and ferroptosis provide valuable implications for cancer therapy and drug resistance.

#### 5.1.4 Pseudouridylation‐supervised tumour immune evasion

Tumour immune evasion, which involves the ability of tumour cells to circumvent immune detection, plays a crucial role in tumour progression and resistance to immunotherapy. Pseudouridylation has been implicated in promoting tumour immune evasion by modifying the tumour microenvironment and disrupting immune surveillance mechanisms. Multiomics analyses have demonstrated a correlation between PUS1‐induced malignancies and immune cell infiltration in NSCLC. Further analysis using tools such as TIMER, ESTIMATE, and IPS suggests that PUS1 expression is associated with immune cell infiltration, particularly showing a strong negative correlation with DC infiltration,^92^ indicating that PUS1 may influence immune cell infiltration by modulating pseudouridylation levels. The tumour microenvironment is a critical determinant of tumour pathogenesis, malignancy, and therapeutic outcomes, including in HCC.^93^ Bioinformatics approaches have revealed that PUSs are linked to various immune cell types within the HCC tumour microenvironment.^94^ While research on the specific relationship between pseudouridylation and tumour immune evasion remains limited, existing studies strongly suggest that dysregulated pseudouridylation contributes significantly to this process.

Pseudouridylation has emerged as a critical factor in tumour initiation and progression, playing an essential role in driving cancer development. Extensive evidence links pseudouridylation to tumourigenesis, metastasis, and poor survival outcomes. Research consistently supports the oncogenic role of pseudouridylation in sustaining cancer hallmarks. Although the role of pseudouridylation in tumour immune escape is still underexplored, it holds significant therapeutic potential. In the context of RNA vaccine development, leveraging pseudouridylation to modulate immune responses could lead to substantial therapeutic breakthroughs. While pseudouridylation is predominantly associated with malignant tumour progression, it is also a conserved process in normal physiological functions, suggesting that its role may vary across different tumour types. A deeper interpretation of the diverse roles of pseudouridylation in cancer could pave the way for new targeted therapies, improving treatment outcomes and enhancing the effectiveness of immunotherapies.

### 5.2 Other diseases

#### 5.2.1 X‐linked dyskeratosis congenita (DC)

Mutations in *DKC1*, which encodes the pseudouridine synthase dyskerin, can lead to DC, a rare hematopoietic and malignant disorder characterised by an increased susceptibility to tumours and premature aging, as well as its variant, Hoyeraal–Hreidarsson syndrome.^95^, ^96^, ^97^ Clinically, DC manifests with nail dystrophy, reticular skin pigmentation, oral leukoplakia, and systemic issues, including bone marrow failure, pulmonary fibrosis, liver disease, and neurological and ocular disorders.^18^, ^98^ Initially, X‐linked DC (X‐DC) was not associated with pseudouridine deficiency but rather attributed to compromised replicative capacity due to impaired telomerase activity caused by shortened telomeres.^99^ However, recent studies have demonstrated defects in rRNA pseudouridylation in patients with X‐DC.^76^ Furthermore, research involving mice with *DKC1* mutations or altered expression suggests that abnormalities in ribosome biogenesis and/or pseudouridylation also contribute to the pathogenesis of DC.^14^, ^100^ Consequently, dyskeratosis congenita is strongly linked to the dysregulation of pseudouridylation.

#### 5.2.2 Neurological and metabolic diseases

Mutations in *PUS3* and *PUS7* are also closely associated with neurological disorders. Individuals with homozygous missense and frameshift deletion mutations in *PUS7* present with intellectual disabilities, microcephaly, speech delay, short stature, and aggressive behaviour. These mutations result in reduced *PUS7* mRNA expression in patient fibroblasts, leading to a decrease in pseudouridylation at position 13 of tRNAs.^101^, ^102^ Similarly, a homozygous truncating mutation in *PUS3* reduces pseudouridylation at positions 38 and 39 in tRNAs, which correlates with intellectual disability.^103^ MLASA, a metabolic disorder, is associated with defects in pseudouridylation, primarily manifesting as exercise intolerance and anaemia.^104^ This rare autosomal recessive disorder, linked to *PUS1* gene mutations, leads to a partial or complete loss of pseudouridylation at positions 27 and/or 28 of tRNAs.^104^ Pseudouridylation also plays a critical role in the pathogenesis of maternally inherited diabetes and deafness (MIDD), where defects hinder the conversion of U55 to Ψ55 in mitochondrial tRNA‐Glu. This alteration disrupts the tertiary structure of mitochondrial tRNA, increasing instability and impairing mitochondrial translation and respiratory function. The subsequent disruption in respiratory patterns enhances reactive oxygen species (ROS) production, which predominantly affects pancreatic beta‐cells, neurons, and cochlear hair cells, leading to the clinical manifestations of MIDD.^105^

#### 5.2.3 Cardiovascular diseases (CVD)

Plasma and urine levels of Ψ have been linked to cardiovascular disease, with elevated plasma Ψ concentrations observed in individuals with heart failure and reduced ejection fraction (HFrEF) compared to healthy controls.^106^, ^107^ This elevation suggests that plasma Ψ, in conjunction with natriuretic peptide levels, could serve as a valuable biomarker for improving the diagnosis of HFrEF. Additionally, studies in patients with Tetralogy of Fallot have shown that the expression of scaRNA1 in right ventricular cardiac tissue is reduced, leading to a corresponding decrease in Ψ levels.^18^, ^52^ These findings highlight the potential of pseudouridylation‐related biomarkers in advancing the diagnosis and understanding of cardiovascular diseases.

Overall, pseudouridine synthases play a critical role in maintaining cellular homeostasis, with their dysfunction being linked to a variety of diseases. While research on the relationship between pseudouridylation and non‐tumour diseases remains largely observational, further exploration into the underlying mechanisms, including gene mutations and changes in post‐transcriptional modifications, is essential. Despite current gaps in understanding, it is evident that pseudouridylation is a key factor in non‐tumour diseases. Additional research is demanded for comprehensively elucidating the pathogenic mechanisms of pseudouridylation in these conditions.

## 6 THE THERAPEUTIC POTENTIAL OF RNA PSEUDOURIDYLATION IN DISEASES

### 6.1 Inhibitors targeting pseudouridine synthases

PUSs play a pivotal role in the pseudouridylation process by both recognising and catalysing the modification of RNA substrates. Dysregulation of these enzymes can disrupt pseudouridylation levels, contributing to various diseases. Notably, aberrations in enzymes such as PUS7, PUS1, PUS10, and DKC1 have been implicated in several disorders.^13^, ^92^, ^108^, ^109^, ^110^, ^111^, ^112^ Despite the established connection between pseudouridylation and disease, the absence of specific inhibitors targeting pseudouridine‐modifying enzymes has hindered the development of targeted therapeutic strategies. Therefore, the identification of specific inhibitors for these enzymes is of great importance for treating diseases resulting from pseudouridylation dysregulation.

Recent studies have made promising advances in targeting PUS7 to suppress tRNA pseudouridylation and inhibit glioblastoma tumourigenesis.^13^ In a virtual screening of 270 000 NCI‐DTP compounds and 4086 FDA‐approved drugs, small molecules that modulate PUS7 activity were identified. Two compounds, C4 and C17, were found to inhibit PUS7‐mediated pseudouridylation and demonstrated efficacy in inhibiting GSC growth both in vitro and in vivo. These inhibitors present potential as therapeutic candidates targeting PUS7 in glioblastoma and potentially other cancers. Furthermore, recent research has explored the Heat shock protein 90 /PUS7/ LIM and SH3 protein 1 (HSP90/PUS7/LASP1) axis in CRC, revealing that inhibitors of HSP90, such as NMS‐E973, also inhibit PUS7‐mediated pseudouridylation. This suggests that HSP90‐mediated regulation of PUS7 could offer a novel therapeutic approach, although these inhibitors do not directly target PUS7. They may serve as viable candidates for modulating PUS7 activity in therapeutic contexts.^108^ The discovery of PUS7 inhibitors represents a significant step forward, offering new opportunities for targeted therapies aimed at correcting the dysregulation of pseudouridylation enzymes in cancer and potentially other diseases. With the development of advanced small molecule screening technologies, the future identification of additional inhibitors targeting PUSs holds promise for expanding the pool of potential therapeutic agents for pseudouridylation‐related diseases.

### 6.2 Inhibitors targeting DKC1

Pyrazofurin, tested as an orotidine 5′‐monophosphate (OMP) decarboxylase inhibitor in cancer clinical trials, demonstrated limited efficacy due to low reaction rates and significant toxicity.^113^ Recent studies, however, have emphasised the carcinogenic role of DKC1, a pseudouridine‐modifying enzyme, in CRC. Notably, Pyrazofurin, when combined with trametinib (a MAP kinase inhibitor), effectively inhibits the growth of CRC cells with elevated DKC1 expression in vitro and in vivo, suggesting potential applications in DKC1‐overexpressing cancers.^112^ Additionally, Pyrazofurin markedly reduced the pseudouridylation activity of DKC1 on selected uridines within 28S rRNA. Previous trials with Pyrazofurin, however, did not assess its impact on the biological activity of human dyskerin, warranting further investigation into its utility in cancers with high DKC1 expression.^113^ However, further research is required to determine whether these inhibitors affect pseudouridylation levels.

Overall, the development of targeted inhibitors for pseudouridine‐modifying enzymes remains in the early stages, but the potential for discovering effective inhibitors is considerable, with virtual screening offering a promising strategy for identifying suitable candidates.

### 6.3 The potential mechanism of 5‐fluorouracil inhibiting pseudouridylation

5‐fluorouracil (5‐FU), a staple for various solid tumour treatment, including CRC, breast cancer, and HCC,^114^, ^115^ has been used for nearly 70 years, though its precise mechanism of action remains debated. Evidence suggests that 5‐FU's primary target may not involve DNA metabolism.^116^, ^117^ In HeLa cells treated with 5‐FU, a significant accumulation of pre‐mRNA has been observed.^118^ Thin‐layer chromatography of snRNA *U2* from these cells revealed the presence of 5‐FU alongside a decrease in pseudouridine levels.^47^ These findings suggest that part of 5‐FU's therapeutic efficacy may stem from its ability to inhibit pre‐mRNA splicing by preventing pseudouridine formation,^47^ offering a novel perspective on its potential as a pseudouridylation inhibitor in future cancer therapies.

### 6.4 RNA therapeutics

The dysregulation of pseudouridylation plays a pivotal role in disease progression and provides valuable insights for RNA‐based therapeutics. Cellular immune surveillance, mediated by Toll‐like receptors (TLRs), retinoic acid‐inducible protein I (RIG‐I), and protein kinase R (PKR), presents a significant barrier to the use of exogenous therapeutic RNA, which is rapidly recognised and eliminated.^119^ However, RNA modified with pseudouridine can evade detection by these immune sensors, allowing it to remain stable and exert therapeutic effects for prolonged periods.^33^ This feature makes pseudouridylation an effective tool in RNA therapeutics. For instance, in vitro transcribed RNA induces an immune response when introduced into HEK293 cells expressing TLR3, TLR7, or TLR8, but the incorporation of pseudouridine mitigates this response.^120^ Furthermore, pseudouridine‐modified 5′‐triphosphate‐capped RNA prevents activation of RIG‐I,^121^, ^122^ highlighting an additional mechanism by which pseudouridine inhibits innate immune activation. Additionally, pseudouridine incorporation into PKR substrates diminishes PKR activation and subsequent translation inhibition compared to undecorated RNA.^33^ In vitro transcriptional reporter RNA containing uridine triggers a rise in mouse interferon‐α levels, whereas fully pseudouridylated reporter RNA suppresses this effect.^74^ Despite the demonstrated success of pseudouridylation in evading immune clearance, further investigation is necessary to fully elucidate its role in RNA therapy. The efficacy of RNA therapy depends not only on immune evasion but also on ensuring the translation of sufficient and functional proteins. While pseudouridine‐modified RNA has been shown to produce more stable and abundant proteins compared to unmodified RNA, the precise mechanisms remain unclear.^72^, ^74^, ^123^ Ongoing studies into mRNA pseudouridylation suggest that its advantages may extend beyond immune evasion to include enhanced mRNA stability, as well as improved translational fidelity and efficiency.

### 6.5 Ψ as a biomarker for cancer

Beyond its therapeutic potential, Ψ and its modifying enzymes also show promise as biomarkers for cancer diagnosis and prognosis.^109^, ^124^, ^125^ This discussion focuses primarily on Ψ itself as a biomarker, rather than its modifying enzymes. Human cells lack enzymes capable of metabolising C‐glycosyl compounds, allowing excessive Ψ to accumulate and be identified in multiple biological fluids, such as plasma, urine, and saliva.^124^ Elevated Ψ levels in the saliva of patients with oral squamous cell carcinoma have been linked to increased RNA degradation rates.^126^ Moreover, Ψ has shown potential as an early diagnostic marker for high‐risk patients. For example, plasma Ψ levels rise in patients with ovarian cancer before clinical diagnosis, suggesting that Ψ dysregulation could serve as an indicator of early‐stage ovarian cancer and as a preclinical biomarker.^127^ In clinical practice, the use of saliva and plasma for molecular diagnostics offers the advantage of relatively simple sample collection.^128^, ^129^ As Ψ becomes more established as a tumour biomarker, detection methods are likely to advance. While traditional approaches like mass spectrometry may be costly, emerging technologies such as molecularly imprinted polymers provide a cost‐effective and practical means of detecting Ψ in body fluids.^130^

**FIGURE 5:**
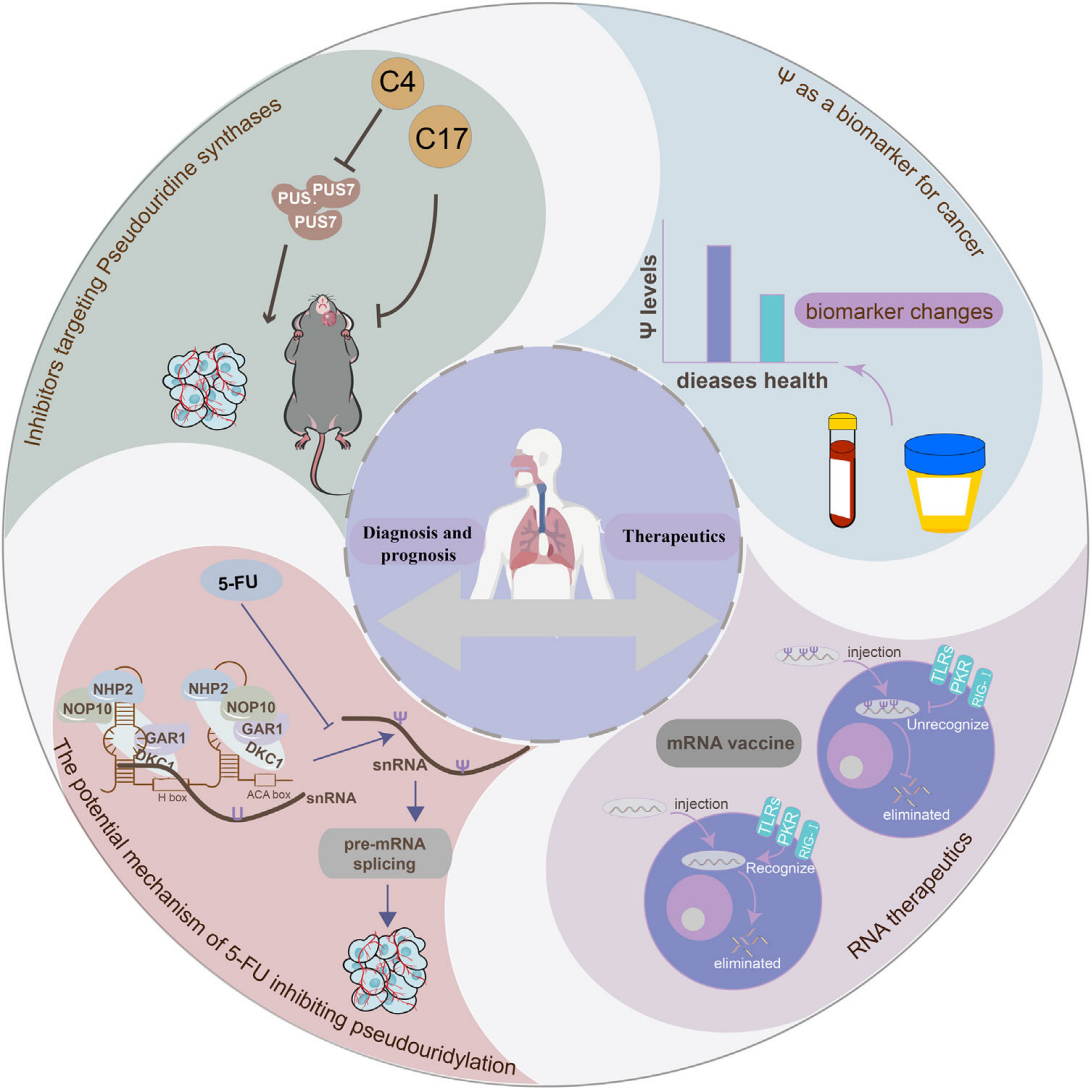
Therapeutic potential of RNA pseudouridylation in diseases. Targeted inhibitors of pseudouridylation enzymes hold promise for treating diseases caused by pseudouridylation dysregulation. For instance, the potential role of 5‐FU as a pseudouridine modification inhibitor in cancer therapy is gaining interest. Ongoing studies suggest that mRNA pseudouridylation may enhance mRNA stability, translation fidelity, and efficiency, extending its therapeutic benefits beyond immune evasion. Additionally, pseudouridine (Ψ) and related proteins are emerging as potential biomarkers for disease prognosis, with significant promise in future diagnostic and prognostic applications. The prominent role of pseudouracil disorders in non‐tumour diseases indicates that targeting pseudouridylation for the treatment of such diseases holds considerable promise for future therapeutic strategies.

In total, proteins involved in pseudouridylation contribute to disease characteristics, and developing inhibitors targeting these proteins can enhance drug diversity and enable personalised treatments. However, while many pseudouridylation inhibitors show promise in cancer therapy, the potential toxicity and side effects need further estimation. Furthermore, given the role of pseudouridylation in tumourigenesis and physiological processes, a comprehensive evaluation of the safety and effectiveness of these inhibitors is critical prior to clinical application. RNA therapy, though promising, faces limitations, but the immune evasion capabilities of pseudouridylation may help mitigate some of these challenges. Additionally, Ψ itself, along with other pseudouridylation‐related proteins, holds significant potential in patient prognosis assessment. As an emerging biomarker, Ψ is expected to play an increasingly prominent role in disease diagnosis and prognosis in the future (Figure 5). While reports on the therapeutic potential of pseudouridylation in non‐tumour diseases are limited, the prominent role of pseudouracil disorders in non‐tumour diseases indicates that targeting pseudouridylation for the treatment of such diseases holds considerable promise for future therapeutic strategies.

## 7 CONCLUSIONS AND PERSPECTIVE

Despite over 70 years of research, the role of pseudouridylation in biological processes remains incompletely understood. Current investigations primarily focus on how pseudouridylation impacts protein translation, with particular emphasis on how modifications of various RNA types influence different stages of protein synthesis. Advances in sequencing technologies have enabled significant progress in mapping pseudouridylation patterns, particularly in rRNA, tRNA, and more recently, mRNA, with a growing focus on non‐coding RNAs. High‐throughput sequencing has been pivotal in charting pseudouridylation distribution at the mRNA level, facilitating deeper insights into mRNA modification.

Pseudouridylation dysregulation has been consistently linked to a range of human diseases, including congenital disorders and various cancers. Enzymes and molecules involved in pseudouridylation play critical roles in the malignant progression of cancers, and inhibitors targeting pseudouridylation have shown promise in slowing cancer progression. These findings underscore the importance of understanding the abnormalities of pseudouridylation in human diseases, offering insights into the development of tumour and non‐tumour disease and potential new therapeutic strategies. Moreover, exploring mechanisms underlying pseudouridylation imbalances—whether shared across tumours or context‐specific—can shed light on the role of the RNA transcriptome in tumourigenesis.^131^

This review provides a comprehensive overview of pseudouridylation, summarising its processes and functions across various RNA types and diseases, while offering valuable insights for future research. Although previous reviews have extensively discussed the biological significance of RNA pseudouridylation,^18^, ^33^, ^132^ this review further elaborates on its roles in tRNA homeostasis and transport, regulation of translation initiation, enhancement of mRNA translation capacity, and other biological implications. Additionally, it summarises the latest technologies for pseudouridine modification, outlining the advantages and limitations of each approach to assist researchers in the field. Notably, this review is the first to systematically explore the mechanisms of pseudouridylation in tumour‐related diseases, incorporating various tumour cell behaviours, thus offering important references for researchers. However, the pseudouridylation process itself remains incompletely defined. Therefore, further in‐depth research is crucial, particularly focused on elucidating the mechanisms and clinical applications of pseudouridylation‐targeted inhibitors, as well as identifying new Ψ readers or reader‐like proteins. Recent studies have identified RNA‐binding proteins, such as Pumilio 2 (PUM2), muscleblind‐like 1 (MBNL1), and methionyl‐tRNA synthetase (MetRS), which interact with RNA based on its pseudouridylation status.^133^, ^134^, ^135^ Additionally, the mechanisms underlying stress‐induced pseudouridylation require further investigation. A deeper understanding of the molecular mechanisms and biological significance of pseudouridylation in disease pathogenesis could pave the way for developing novel treatment strategies.

## AUTHOR CONTRIBUTIONS

Kai Li, Heng Zhou, and Yanshu Li conceived the structure of the manuscript. Shiheng Jia, Xue Yu, and Na Deng drafted the manuscript. Kai Li, Heng Zhou, and Yanshu Li supervised the revision of the manuscript. Shiheng Jia, Xue Yu, Na Deng, Ziming Gao, and Chen Zheng revised the manuscript. Shiheng Jia, Xue Yu, Na Deng, Mingguang Ju, Yixiao Zhang, Fanglin Wang, and Heng Zhou prepared the figures. All authors read and approved the final manuscript.

## CONFLICT OF INTEREST STATEMENT

The authors declare no competing interests.

## ETHICS STATEMENT

The authors declare no conflicts of interest.

## CONSENT FOR PUBLICATION

All authors consent to publication.

## Data Availability

The data that support the findings of this study are available from the corresponding author upon reasonable request.
